# Supplementary material for: SNVPhyl: a single nucleotide variant phylogenomics pipeline for microbial genomic epidemiology
Source: Microb Genom. 2017 Jun 8;3(6):e000116. doi: 10.1099/mgen.0.000116 (PMC5628696; doi:10.1099/mgen.0.000116)

Supplementary Materials/Methods

SNVPhyl Parameters

Unless otherwise specified, SNVPhyl analyses for this study were run using the SNVPhyl workflow version 1.0, with paired-end sequence reads, and with parameters:

- Variant quality parameters
  - Minimum coverage = 10X
  - Relative SNV abundance = 0.75
  - Minimum mean mapping quality = 30
- SNV density filtering
  - Window size = 20
  - SNV threshold = 2
- Repeat identification
  - Minimum length = 150
  - Minimum percent identity = 90%

All other parameters were left at the default settings as recorded in the SNVPhyl Galaxy workflow.

Supplementary Figures and Tables

Figure S1 was constructed using the phylogenetic trees produced in the "SNV density filtering evaluation" section of the main manuscript. The tree produced from the "truth" set of SNVs identified using Gubbins is used as a reference tree and is annotated as "Original alignment" in the figure. This reference tree is compared to all other phylogenetic trees from each SNV-density filtering scenario. The comparisons were made using the phytools [34] package in R.

Figure S2 was constructed using the phylogenetic trees produced in the "Parameter optimization" section of the manuscript. The phylogenetic trees were evaluated for concordance with the epidemiological data according to the conditions: 1) all outbreak isolates group monophyletically, and 2) the maximum SNV distance between any two isolates within an outbreak clade must be less than 5 SNVs. These conditions were tested and the figure constructed using the APE [37] package within R.

Table S1 was constructed from previously published strain and accession identifiers [32]. These were mapped to sequencing run accession identifiers using SRAdb [35].

Table S2 was constructed using information obtained from a previous study [36] along with information from the project SRP067504 under the NCBI Sequence Read Archive.

Table S3 was constructed using the SNVs identified by SNVPhyl from the simulated dataset. The copy numbers covering each position were determined by aligning each variant genome to the reference

genome using the MUMmer [25] software package. In particular, the command “show-snps” was used to extract the number of alignments, and so copies, covering each position.

Supplementary Figures and Tables

Figure S1.

Figure S2.

Table S1.

Separate file **Table_S1.xlsx**

Table S2.

Separate file **Table_S2.xlsx**

Table S3.

Separate file **Table_S3.xlsx**

Figure S1

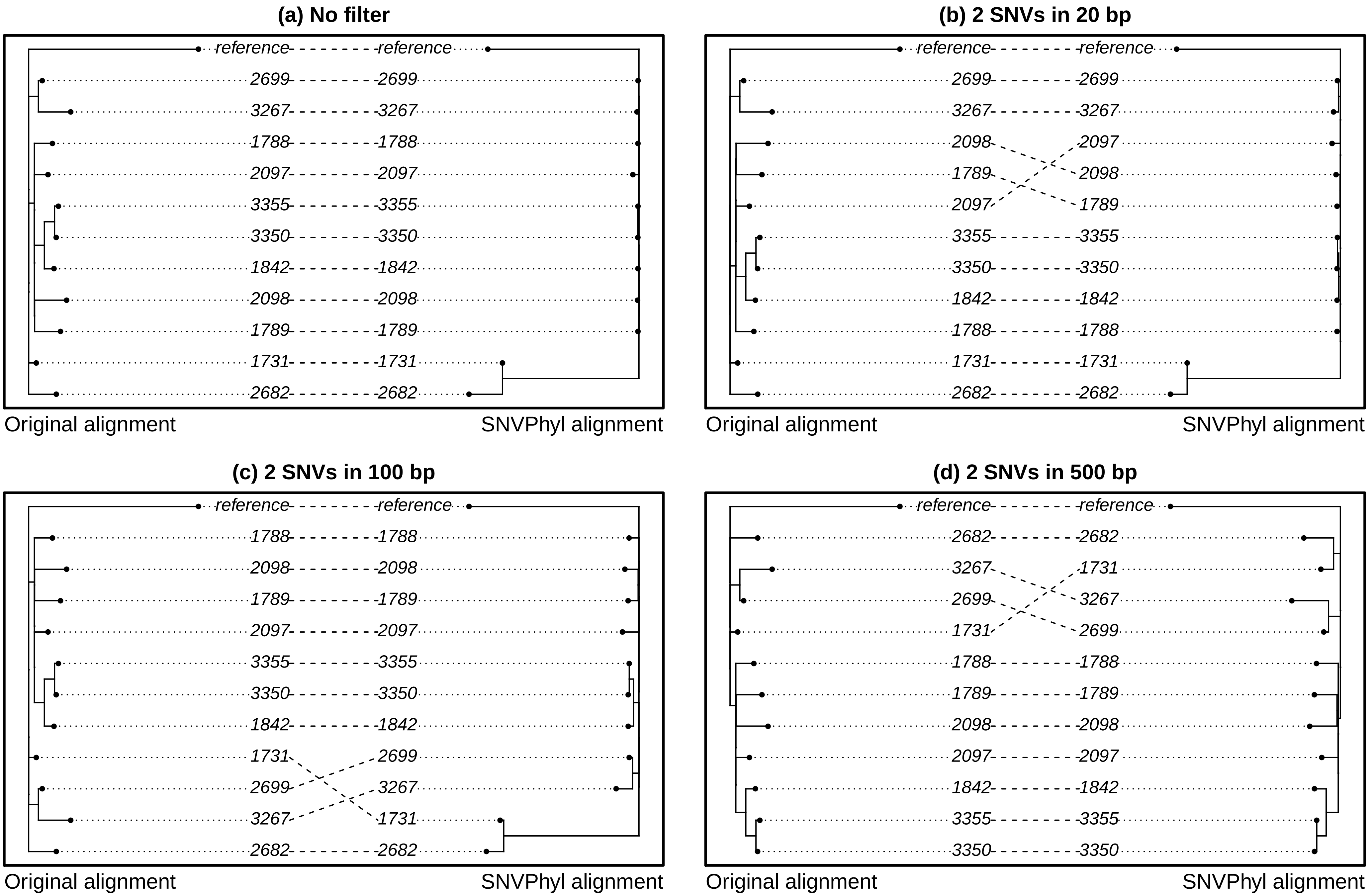

(a) No filter
reference
2699
3267
1788
2097
3355
3350
1842
2098
1789
1731
2682
reference
2699
3267
1788
2097
3355
3350
1842
2098
1789
1731
2682
Original alignment
SNVPhyl alignment
(b) 2 SNVs in 20 bp
reference
2699
3267
2098
1789
2097
3355
3350
1842
1788
1731
2682
reference
2699
3267
2097
2098
1789
3355
3350
1842
1788
1731
2682
Original alignment
SNVPhyl alignment
(c) 2 SNVs in 100 bp
reference
1788
2098
1789
2097
3355
3350
1842
1731
2699
3267
2682
reference
1788
2098
1789
2097
3355
3350
1842
2699
3267
1731
2682
Original alignment
SNVPhyl alignment
(d) 2 SNVs in 500 bp
reference
2682
3267
2699
1731
1788
1789
2098
2097
1842
3355
3350
reference
2682
1731
3267
2699
1788
1789
2098
2097
1842
3355
3350
Original alignment
SNVPhyl alignment

Figure S1

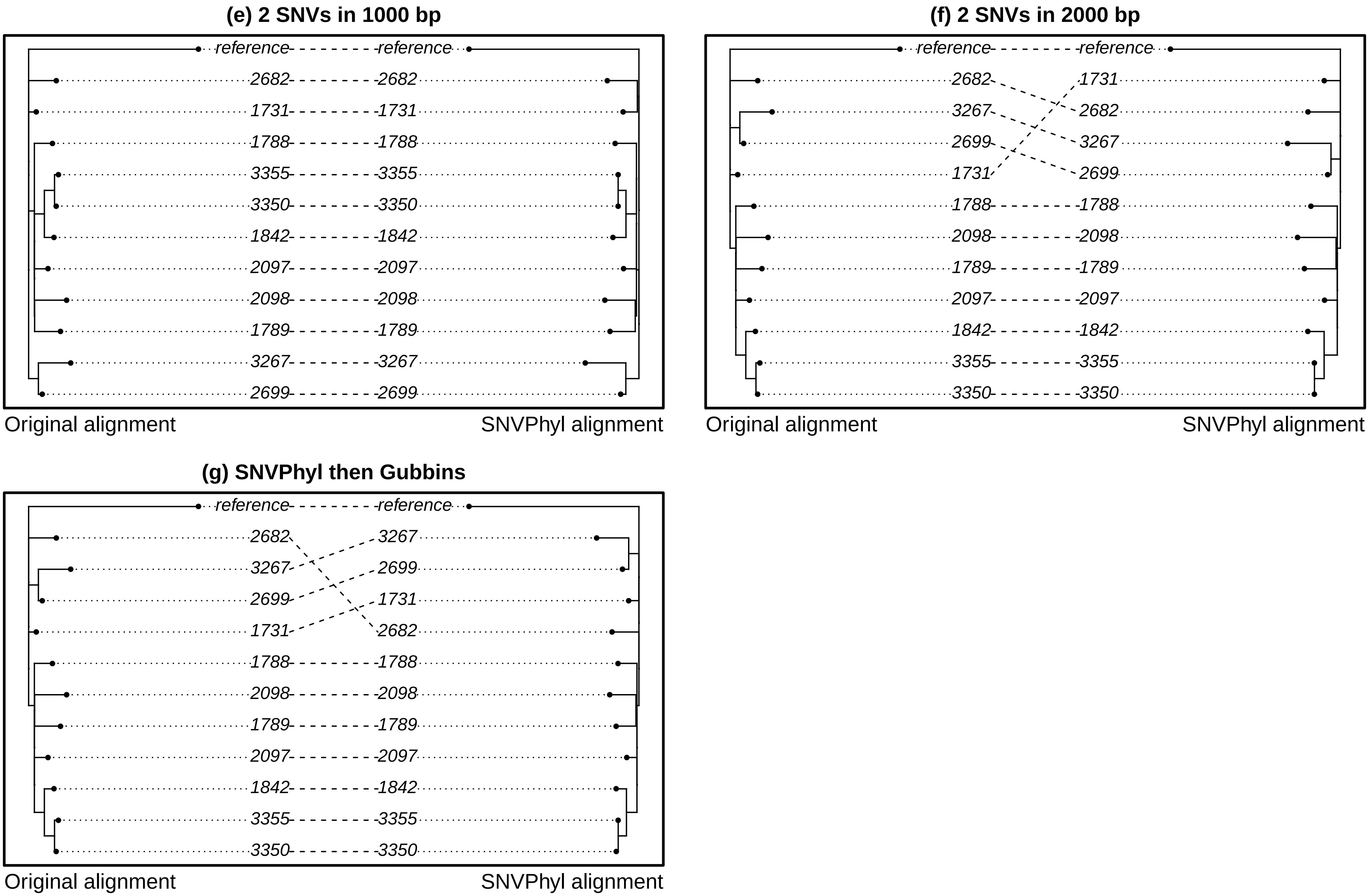
(e) 2 SNVs in 1000 bp
reference
2682
1731
1788
3355
3350
1842
2097
2098
1789
3267
2699
reference
2682
1731
1788
3355
3350
1842
2097
2098
1789
3267
2699
Original alignment
SNVPhyl alignment
(f) 2 SNVs in 2000 bp
reference
2682
3267
2699
1731
1788
2098
1789
2097
1842
3355
3350
reference
1731
2682
3267
2699
1788
2098
1789
2097
1842
3355
3350
Original alignment
SNVPhyl alignment
(g) SNVPhyl then Gubbins
reference
2682
3267
2699
1731
1788
2098
1789
2097
1842
3355
3350
reference
3267
2699
1731
2682
1788
2098
1789
2097
1842
3355
3350
Original alignment
SNVPhyl alignment

Figure S2

(a) Minimum Coverage

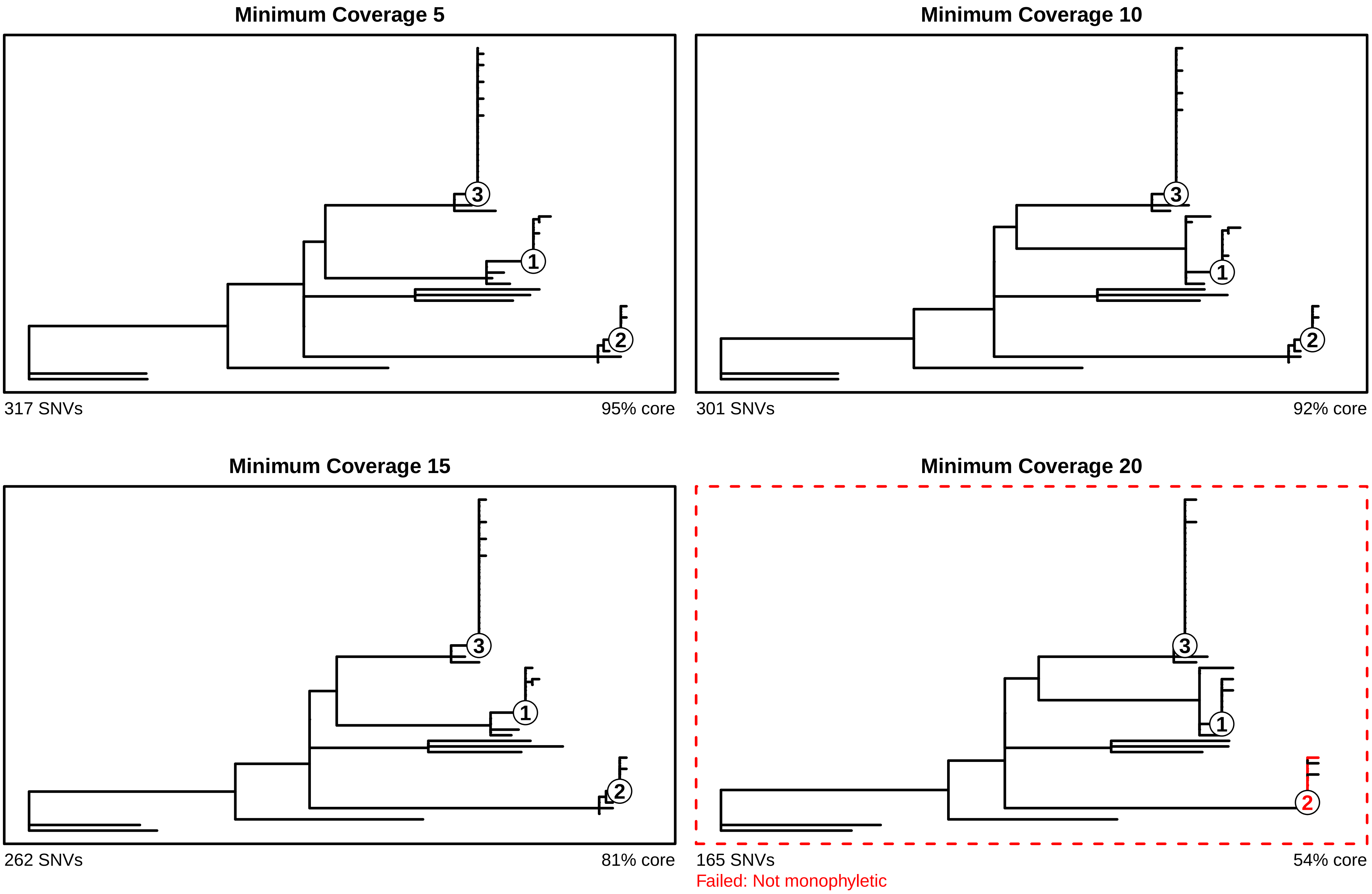

Figure S2

(b) Subsample coverage level

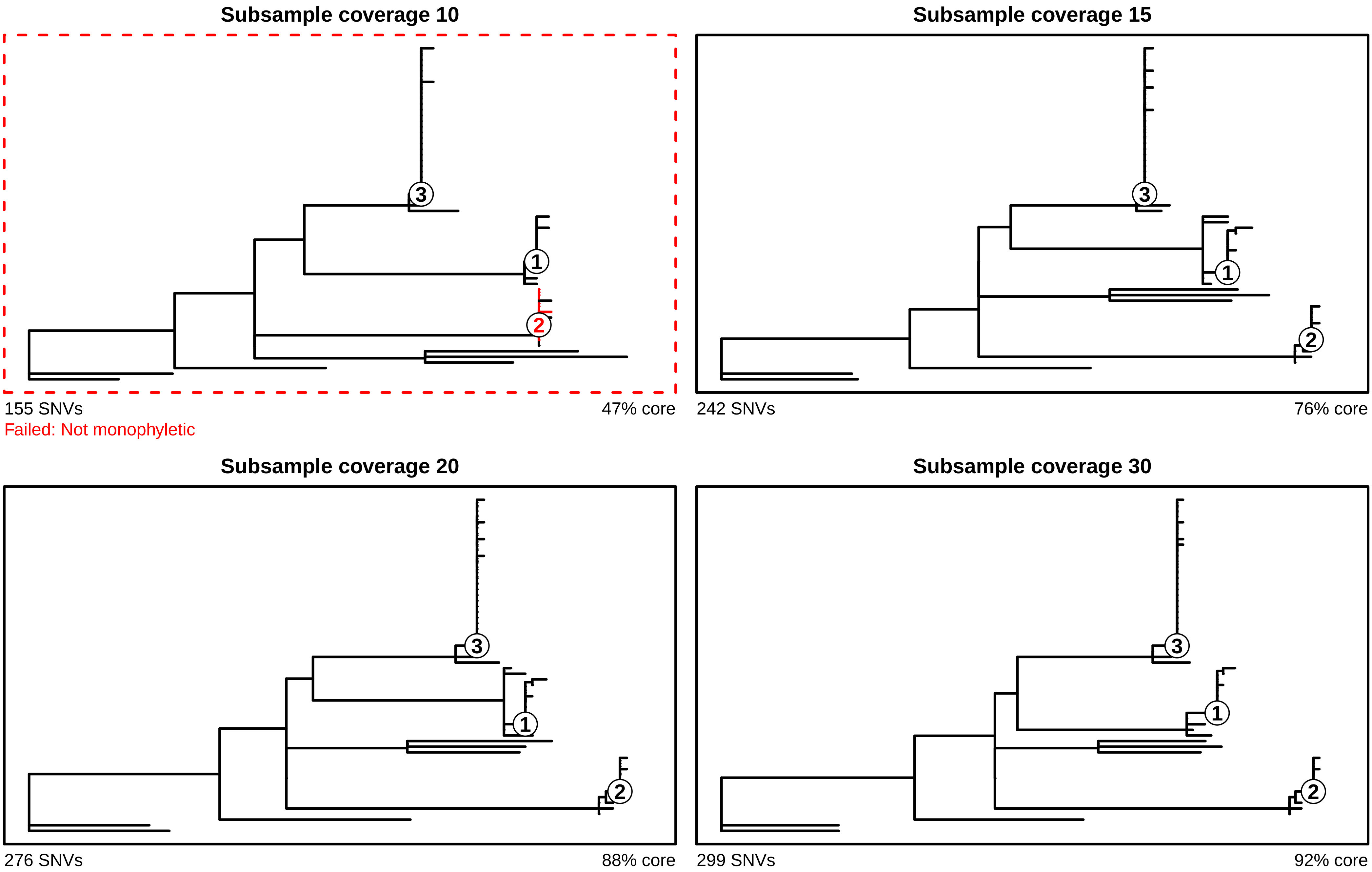

Figure S2
(c) Relative SNV Abundance

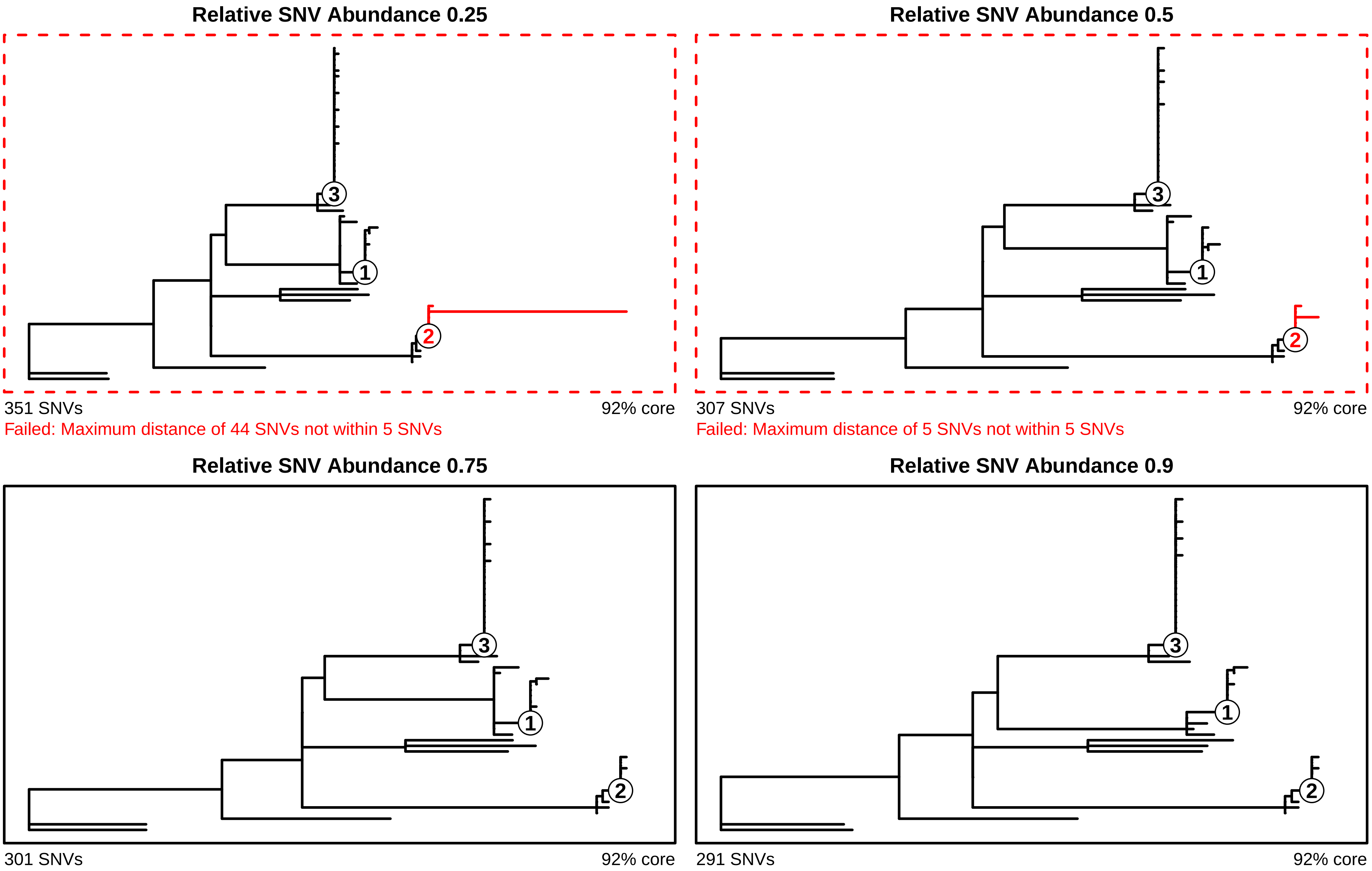

Figure S2
(d) Contamination

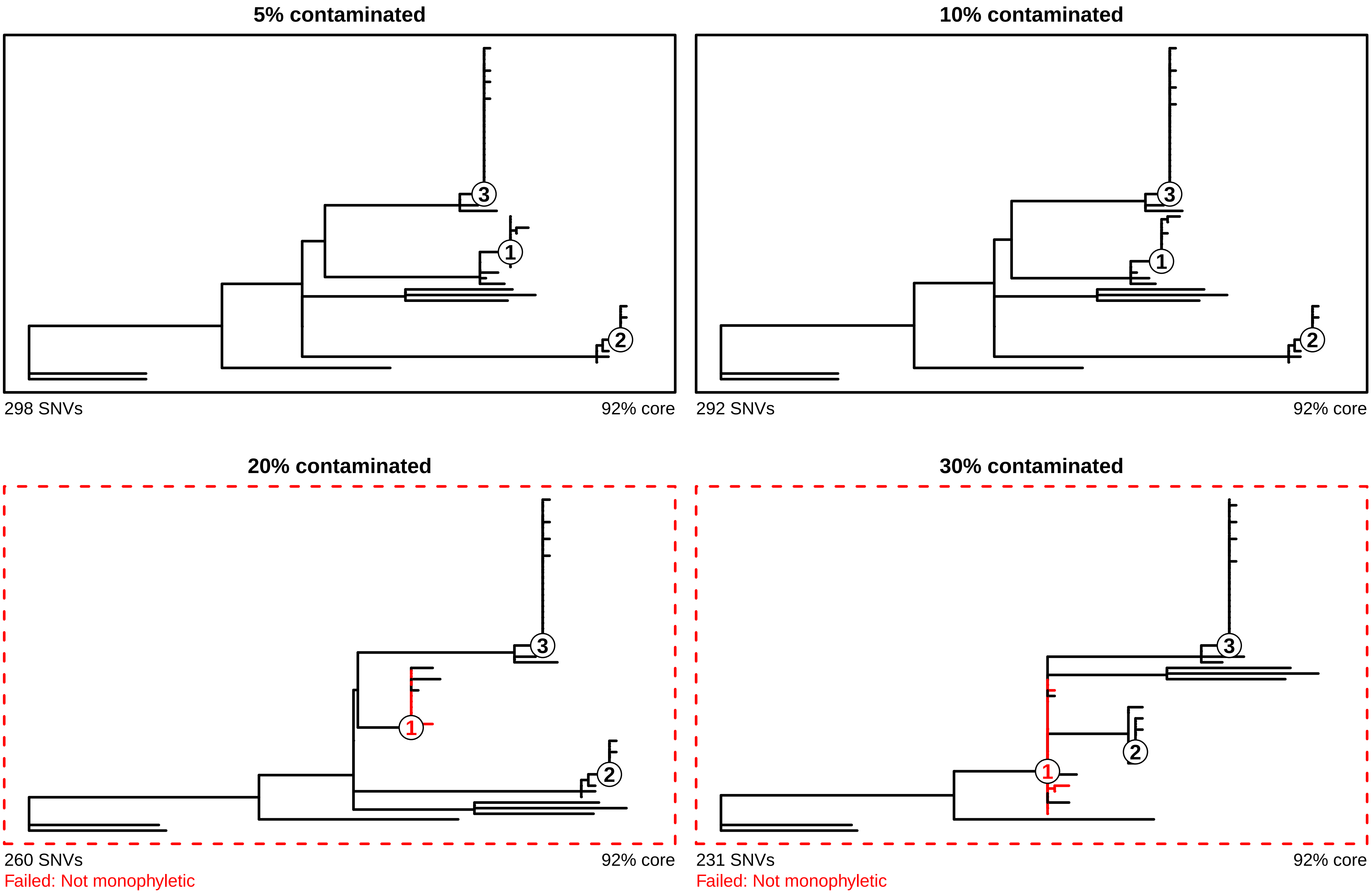

Supplement: Supplementary File 1 [file mgen-3-116-s001.pdf]
